# Targeting Osteosarcoma: The Dual Action of Halogenated Boroxine and Cerium Oxide Nanoparticles

**DOI:** 10.3390/ijms26209837

**Published:** 2025-10-10

**Authors:** Nikolina Tomic, Sahra Esmkhani, Jamila Bayramova, Ahmet Dinc, Ahsen Morva, Belmina Saric Medic, Jasmin Ramic, Naida Lojo-Kadric, Maria Gazouli, Borivoj Galic, Lejla Pojskic, Hilal Yazici

**Affiliations:** 1 Institute for Genetic Engineering and Biotechnology, University of Sarajevo, Zmaja od Bosne 8, 71000 Sarajevo, Bosnia and Herzegovinabelmina.saric@ingeb.unsa.ba (B.S.M.); jasmin.ramic@ingeb.unsa.ba (J.R.); naida.lojo@ingeb.unsa.ba (N.L.-K.); 2 TÜBİTAK Marmara Research Center, Climate Change and Life Sciences, Biotechnology Research Group, 41470 Kocaeli, Türkiye; sahra.esmkhaniyouvalari@ogr.iu.edu.tr (S.E.); jamila.bayramova@ogr.iu.edu.tr (J.B.); ahmet_dinc47@hotmail.com (A.D.); ahsen.morva@tubitak.gov.tr (A.M.); 3 Division of Cancer Genetics, Department of Basic Oncology, Oncology Institute, Istanbul University, 34093 Istanbul, Türkiye; 4 Institute of Graduate Studies in Health Sciences, Istanbul University, 34093 Istanbul, Türkiye; 5 Department of Molecular Biology and Medical Genetics, Scientific Research Center, Azerbaijan Medical University, AZ1022 Baku, Azerbaijan; 6 Transplant Immunology Research Center of Excellence TIREX, School of Medicine, Koc University, 34010 Istanbul, Türkiye; 7 Laboratory of Biology, Department of Basic Medical Sciences, Medical School, National and Kapodistrian University of Athens, 11725 Athens, Greece; mgazouli@med.uoa.gr; 8 Medical Physics Unit, Department of Radiology, Medical School, National and Kapodistrian University of Athens, Attikon University Hospital, 11725 Athens, Greece; 9 Faculty of Science, University of Sarajevo, Zmaja od Bosne 33-35, 71000 Sarajevo, Bosnia and Herzegovina

**Keywords:** osteosarcoma, halogenated boroxine, cerium oxide nanoparticles

## Abstract

Current standard treatments for osteosarcoma have not been changed for decades and have limited and variable success. The advancement of precision medicine technologies, along with the drug-repurposing and fast drug-screening methodologies available, has opened new avenues for the development of more effective therapeutic strategies. In this study, we evaluated the effectiveness of halogenated boroxine (HB) and dextran-coated cerium oxide nanoparticles—DexCeNPs (SD2)—in an in vitro osteosarcoma model. Both agents were tested individually and in combination. The research encompassed assessments of treatment-related cytotoxicity and cell viability, oxidative stress, and apoptotic and necrotic responses, as well as the effects on 3D spheroid models. The results demonstrated that the effects of HB and SD2 were strongly influenced by the dose, exposure time, and cell type. Both exhibited distinguished antitumor activity through cytotoxicity and specific reactive oxygen species (ROS) induction. The combined treatment produced modulated responses that were dependent on the treatment ratio and cell line, suggesting potential synergistic or selective interactions. Notably, the outcomes of the analysis conducted in 3D models revealed reduced toxicity toward non-tumor cells. These findings suggest the improved efficacy of HB and SD2 used in combination as a selective and novel antitumor strategy and underscore the need for further mechanistic studies at the transcriptomic and proteomic levels to elucidate the underlying pathways and clarify the mechanisms of action.

## 1. Introduction

Osteosarcoma is the predominant malignant bone tumor affecting mainly children and adolescents and is characterized by a strong propensity for metastasis and recurrence [1,2,3]. Current standard therapies have remained largely unchanged for nearly fifty years, typically combining surgery with multi-agent chemotherapy, most often high-dose doxorubicin, methotrexate, and cisplatin [4]. Although these regimens have improved outcomes, their effectiveness is limited by severe side effects and the development of drug resistance. Combination therapies have demonstrated enhanced efficacy and remain a promising strategy to overcome these limitations [5]. However, the survival rates remain suboptimal due to tumor aggressiveness and chemotherapy resistance [1]. Therefore, there is an urgent need for novel therapeutic approaches that can effectively target tumor heterogeneity and resistance while minimizing undesired side effects.

Boron and boronic acid compounds have long been used in the development of therapies for various diseases, including cancer [6,7,8]. Boron neutron capture therapy (BNCT) has been an emerging strategy in osteosarcoma therapy lately [9,10,11]. Notably, boronic acid used in BNCT significantly reduced osteolysis and the tumor burden in osteosarcoma-bearing rat models, with studies also demonstrating its selective accumulation in tumor tissue [11].

Halogenated boroxine K_2_(B_3_O_3_F_4_OH) (HB), a product of the cyclic anhydride of boric acid, is patented as a substance with potential antitumor and antiproliferative effects on skin lesions [12,13,14]. In a series of in vitro and in vivo studies on the antitumor potential of HB, its tumor-suppressive effects on various types of tumors have been observed across a range of HB concentrations with minimal effects on non-malignant cells [15,16,17,18,19,20,21,22]. Results of the research on its effect suggest that it acts as a pro-apoptotic agent in leukemia cells [19,20] and modulates metabolic phenotype and autophagy in human bladder carcinoma cells [21]. Still, its mechanism of action has not been fully elucidated, and its effects on bone tumors have not yet been investigated.

Significant improvements in developing selective tumor-targeting drugs were achieved with the advances in nanotechnology and knowledge of cancer biology [2]. Several nanomedicine formulations have even been clinically approved in recent decades [23]. Their growing use is driven by multifunctional capabilities in drug delivery, diagnosis, and imaging, along with advantages such as an extended circulation time, improved tissue accumulation, and reduced toxicity [23]. Among nanomaterials, cerium oxide nanoparticles (CeNPs) have attracted significant attention due to their unique redox properties, high biocompatibility, and potential biomedical applications. CeNPs exhibit self-regenerative redox activity, enabling dual antioxidant and pro-oxidant effects that regulate ROS levels in biological systems [24]. By mimicking ROS-related enzymes, CeNPs protect normal cells from oxidative stress while inducing cancer cell death, enhancing tumor sensitization for various therapies. Additionally, CeNPs are used as nanocarriers to improve drug delivery and in combination with other drugs to achieve synergistic anticancer effects [24].

In the context of osteosarcoma, CeNPs have shown promising anticancer activity, with selective cytotoxicity enhanced under acidic conditions that mimic the tumor microenvironment [25]. Additionally, there has been a study indicating that higher dextran coating concentrations can improve the therapeutic efficacy of CeNPs while minimizing the toxicity to healthy bone cells [26]. These properties position dextran-coated CeNPs (Dex-CeNPs) as a potential candidate for targeted osteosarcoma treatment.

The strategy of combining or co-delivering multiple therapeutic agents is increasingly employed in cancer research to maximize efficacy through synergistic, additive, or potentiating effects [27,28]. While HB has shown selective antitumor activity in various cancer models, its effectiveness against osteosarcoma remains unknown. On the other hand, as noted above, Dex-CeNPs have also exhibited selective antitumor effects, particularly in the osteosarcoma model. Therefore, the present study aimed to evaluate the cytotoxic and oxidative effects of HB and recently described Dex-CeNPs [29] on osteosarcoma cells. Additionally, the combined effects of HB and Dex-CeNPs were assessed on osteosarcoma and osteoblast cell lines to explore their potential as a novel combinatorial therapeutic approach. To enhance the physiological relevance of these findings, in addition to standard 2D cultures, 3D tumor spheroid models were employed, as they better represent the tumor microenvironment. Thus, evaluating HB, Dex-CeNPs, and their combination in spheroid cultures provided deeper insight into their therapeutic potential.

## 2. Results

The results are presented in the same order as the experiment was conducted. First, the cytotoxicity and ROS profile of two testing treatments were established for all used cell lines. After that an apoptosis profile for single and combined treatment replications in osteosarcoma and non-tumor cell lines was carried out. The relative cell growth in the treated tumor and non-tumor cell lines after the single and combined HB and SD2 treatments was assessed in 3D spheroid models.

### 2.1. Physicochemical Properties

A detailed physicochemical characterization of SD2 was conducted in the study published before [29]. As it is described in that paper, UV–Vis spectrophotometry showed a peak for SD2 at approximately 287 nm, the hydrodynamic diameter was 100 nm (25% ratio), the zeta potential was slightly negative, and the XPS analysis revealed a higher concentration of Ce^3+^, with a Ce^3+^/Ce^4+^ ratio of 1.45. All these characteristics were confirmed in our study, as well for each batch of newly synthesized SD2.

### 2.2. The Cytotoxicity and ROS Profile Measurements

#### 2.2.1. The Cytotoxicity and ROS Profiles of HB on the Osteosarcoma Cell Lines

**Figure 1:**
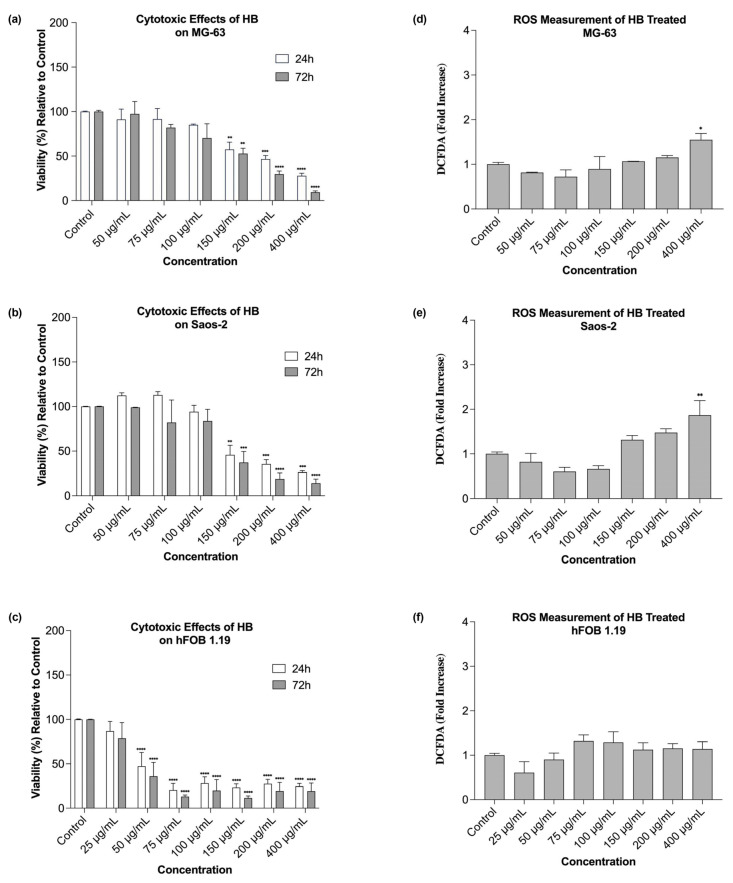
Cytotoxic effects of a series of HB concentrations on the (**a**) MG-63, (**b**) Saos-2, and (**c**) hFOB 1.19 cell lines after 24 h and 72 h of treatment. Dose- and time-dependent variabilities were observed across the tested cell lines. The reactive oxygen species (ROS) generation induced by the same HB concentrations in the (**d**) MG-63, (**e**) Saos-2, and (**f**) hFOB 1.19 cell lines after 24 h of treatment was found to be variable across the cell lines. Statistical significance is indicated as * *p*  <  0.05, ** *p*  <  0.01, *** *p*  <  0.001, and **** *p*  <  0.0001.

**Figure 2:**
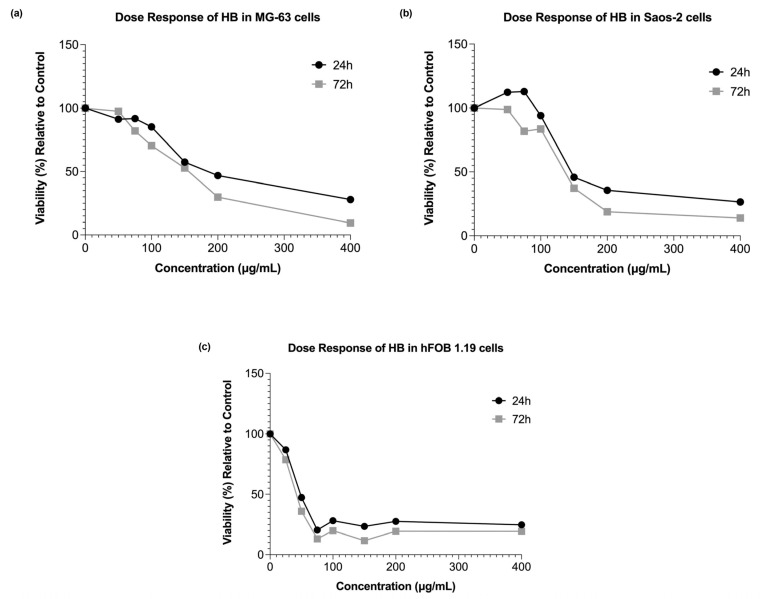
Effect of series of HB concentrations on cell viability in (**a**) MG-63, (**b**) Saos-2, and (**c**) hFOB 1.19 cell lines.

HB demonstrated dose-dependent and time-dependent cytotoxic effects on the MG-63 osteosarcoma cells. Higher concentrations (150 µg/mL, 200 µg/mL, and 400 µg/mL) significantly decreased the cell viability at both 24 and 72 h post-treatment (Figure 1a and Figure 2a). In terms of the exposure duration, the cells treated with the lowest concentration (50 µg/mL) exhibited a slight increase in the viability at 72 h, suggesting a potential recovery effect. Conversely, the cells exposed to higher concentrations showed a continued decline in viability over time. Similarly, in the Saos-2 cells, higher HB concentrations (150 µg/mL, 200 µg/mL, and 400 µg/mL) induced a statistically significant dose-dependent reduction in the cell viability at both time points, mirroring the trend observed in the MG-63 cells. Interestingly, lower concentrations (50 µg/mL and 75 µg/mL) exhibited inhibitory effects only at 72 h of incubation, with measurable declining viability (Figure 1b and Figure 2b). The cytotoxic response was more pronounced in the non-tumor hFOB 1.19 cells compared with the osteosarcoma lines. This prompted the inclusion of a lower concentration (25 µg/mL), which was the only dose that did not significantly reduce the viability at either time point. However, the response in the hFOB 1.19 cells did not display a strictly dose-dependent pattern, e.g., 200 µg/mL had a milder effect than 75 µg/mL and 100 µg/mL. The impact of increasing the HB concentrations appeared more linear than exponential, as depicted in the bar charts and dose response curve (Figure 1c and Figure 2c).

Depending on the cytotoxicity profile observed at 24 h post-treatment and reported above, HB’s half-maximal inhibitory concentrations (IC_50_) were calculated. The HB IC_50_ values for the osteosarcoma cell lines were similar to each other (203 µg/mL for the MG-63 and 192 µg/mL for the Saos-2), while the value for the non-tumor cell line hFOB 1.19 was notably lower (57 µg/mL).

The effects of HB treatment on the ROS production varied across the concentrations. In both the osteosarcoma cell lines, MG-63 and Saos-2, higher concentrations of HB led to increased ROS levels (Figure 1c,d), with the elevation at 400 µg/mL reaching statistical significance (*p*-values of 0.0409 and 0.098 for the MG-63 and Saos-2, respectively). Interestingly, lower concentrations (50 µg/mL, 75 µg/mL, and 100 µg/mL) resulted in reduced ROS levels compared with the untreated controls. In the hFOB 1.19 cell line, all concentrations—except 25 µg/mL and 50 µg/mL—elicited an increase in the oxidative stress relative to the control cells. However, these changes were not statistically significant (Figure 1f).

#### 2.2.2. The Cytotoxicity and ROS Profiles of SD2 on the Osteosarcoma Cell Lines

**Figure 3:**
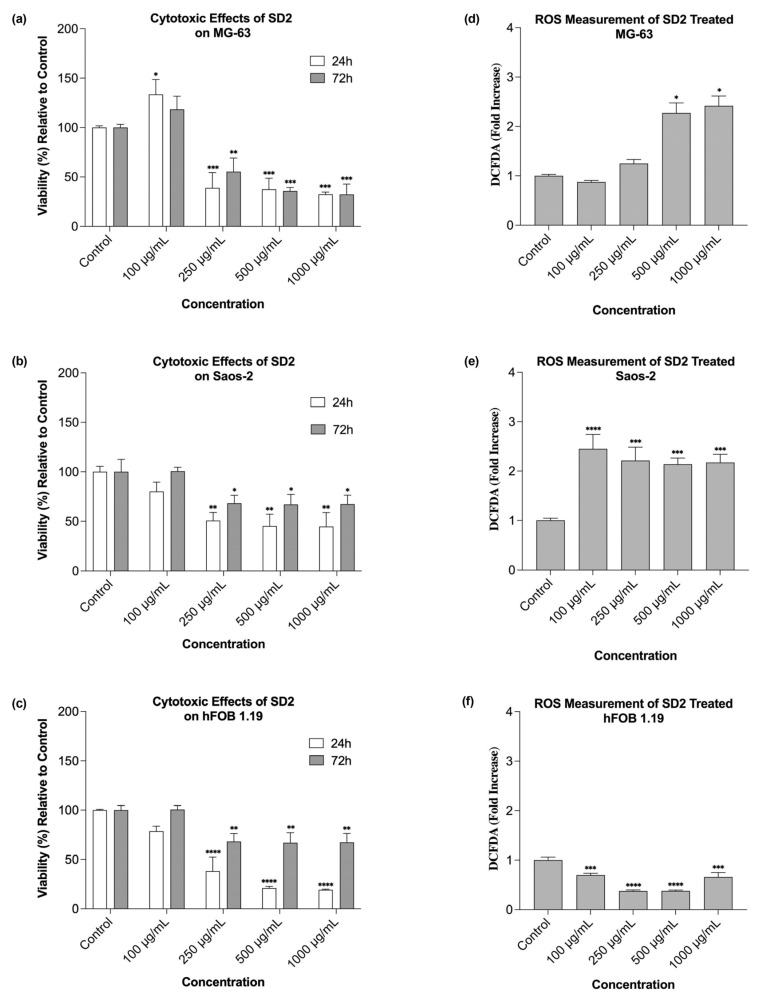
Cytotoxic effects of a series of SD2 concentrations on the (**a**) MG-63, (**b**) Saos-2, and (**c**) hFOB 1.19 cell lines after 24 h and 72 h of treatment. Dose- and time-dependent variabilities were observed across the tested cell lines. The reactive oxygen species (ROS) generation induced by the same SD2 concentrations in the (**d**) MG-63, (**e**) Saos-2, and (**f**) hFOB 1.19 cell lines after 24 h of treatment was found to be variable across the cell lines. Statistical significance is indicated as * *p*  <  0.05, ** *p*  <  0.01, *** *p*  <  0.001, and **** *p*  <  0.0001.

**Figure 4:**
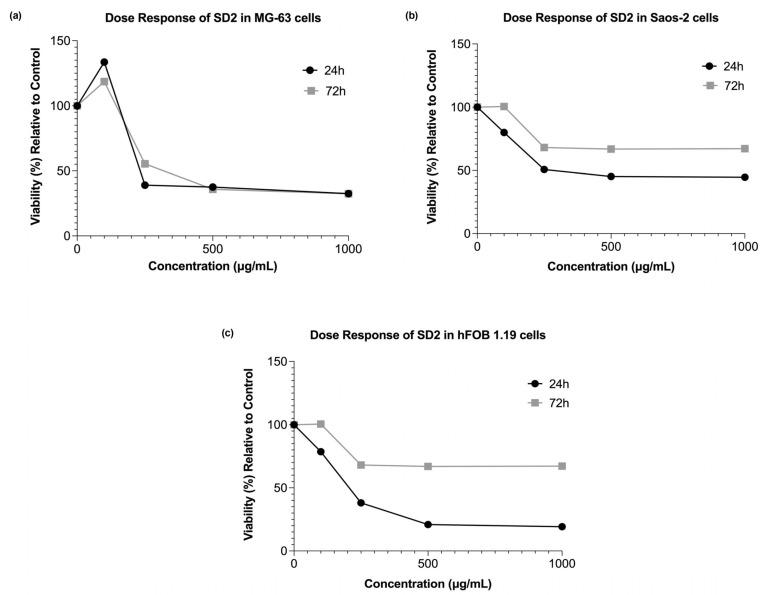
Effect of series of SD2 concentrations on cell viability in (**a**) MG-63, (**b**) Saos-2, and (**c**) hFOB 1.19 cell lines.

SD2 exhibited cytotoxic effects in all three cell lines. In the MG-63 cells, all tested concentrations except 100 µg/mL resulted in a significant decrease in the cell viability at both time points compared with the control (Figure 3a). Interestingly, the treatment with 100 µg/mL led to an increase in the viability, although this effect was less pronounced after 72 h than at 24 h (Figure 4a). The cells treated with 250 µg/mL showed a slight recovery in viability after 72 h, a trend not observed with other concentrations, where extended exposure generally led to a slight decline in the cell viability. The cytotoxic response of the Saos-2 cells to SD2 differed from that observed in the MG-63 cells. At 24 h, a dose-dependent variability in the viability was noted, where higher concentrations produced comparable reductions in the cell viability (Figure 3b and Figure 4b). These higher concentrations significantly decreased the viability at both time points. However, the longer exposure of 72 h appeared to result in a recovery in the viability, suggesting a possible cellular adaptation over time. The cytotoxic effects of SD2 on the hFOB 1.19 cells appeared to be dose-dependent at the 24 h time point, where higher concentrations (250, 500, and 1000 µg/mL) significantly reduced the cell viability compared with the control (Figure 3c). Interestingly, the cells treated with all concentrations showed a potential recovery effect, as the viability increased after 72 h compared with 24 h, although it remained significantly decreased relative to the control (Figure 3c and Figure 4c).

In summary, SD2 induced significant dose- and time-dependent cytotoxicities in the MG-63 and Saos-2 cells starting from 250 µg/mL. The hFOB 1.19 cells were more sensitive to SD2, where they showed a marked decrease in the viability from 250 µg/mL, especially at 72 h (*p* < 0.0001). The SD2 IC_50_ values were calculated based on its cytotoxic profile generated 24 h post-treatment, which are described in the results above. Those values were 132 µg/mL for the MG-63 cell line, 222 µg/mL for the Saos-2, and 142 µg/mL for the hFOB 1.19.

ROS analysis revealed that SD2 significantly elevated ROS production in MG-63 cells at 500 and 1000 µg/mL (*p* < 0.05) (Figure 3d). Saos-2 cells showed a pronounced increase in the ROS levels, even at the lowest concentration (100 µg/mL), with the most significant changes observed at 250 µg/mL (*p* < 0.0001), indicating strong oxidative stress (Figure 3e). In contrast, the hFOB 1.19 cells demonstrated a notable decrease in the ROS generation across all SD2 concentrations, with statistically significant reductions compared with the control (*p* < 0.001 to *p* < 0.0001) (Figure 3f). These findings suggest that SD2 exerted cytotoxic effects on both the cancerous and non-cancerous osteoblast-like cells, potentially via ROS induction in the tumor cells but through different mechanisms in the normal cells. SD2 significantly increased the ROS production in the MG-63 and Saos-2 cells, where the Saos-2 showed strong oxidative responses, even at low concentrations. In contrast, the ROS levels were significantly reduced in the hFOB 1.19 cells at all SD2 doses, suggesting that SD2 induced cytotoxicity through ROS-dependent mechanisms in the cancer cells and ROS-independent pathways in the normal cells.

#### 2.2.3. Time- and Dose-Dependent Efficacies of HB and SD2 Co-Treatment Through Cytotoxicity Analysis and ROS Profile

**Figure 5:**
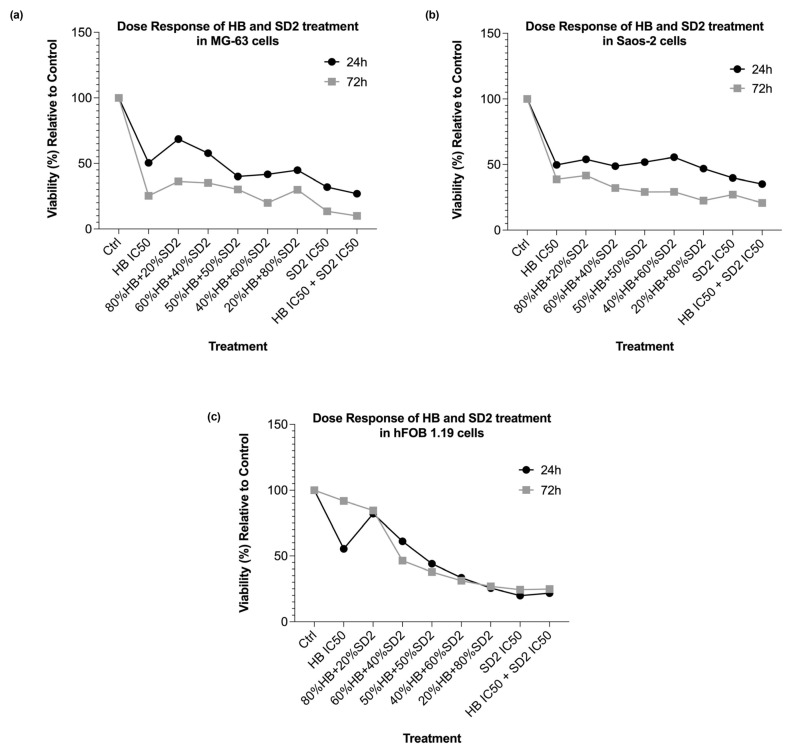
Effects of HB and SD2 treatment on cell viability in (**a**) MG-63, (**b**) Saos-2 and (**c**) hFOB 1.19 cell lines.

**Figure 6:**
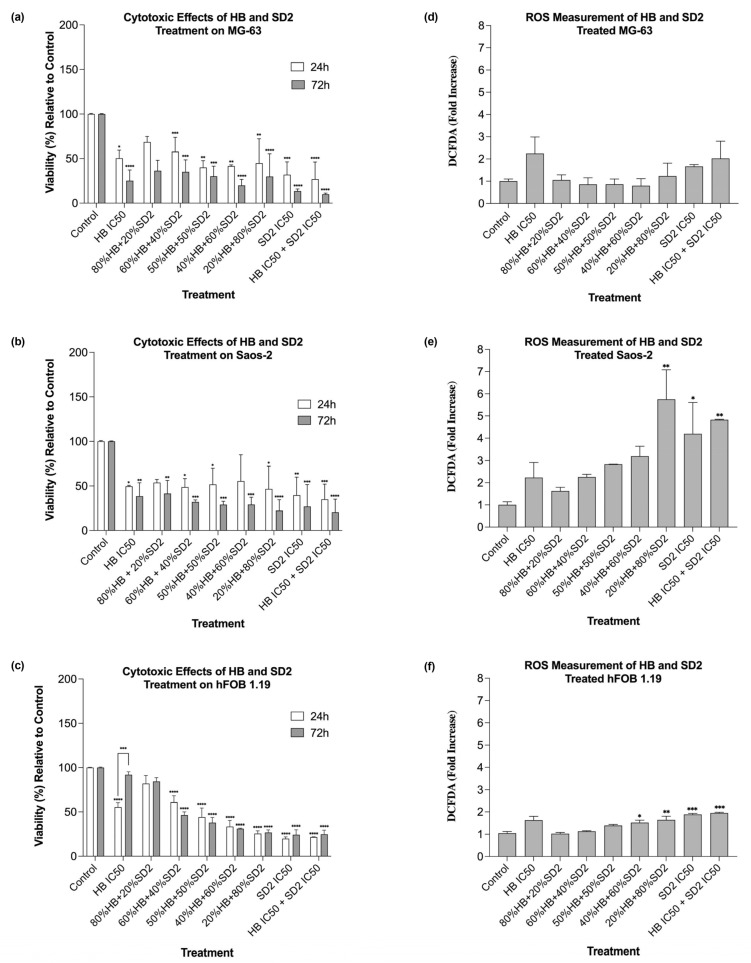
Effects of halogenated boroxine (HB) and Dex-CeNPs (SD2) co-treatment on the cell viability in the MG-63 (**a**), Saos-2 (**b**), and hFOB 1.19 (**c**) cell lines at 24 h and 72 h. Different HB and SD2 combinations showed variable effects. The reactive oxygen species (ROS) generation profile following 24 h treatment was cell-line-specific and variable across treatment combinations in the MG-63 (**d**), Saos-2 (**e**), and hFOB 1.19 (**f**) cell lines. Statistical significance is indicated as * *p*  <  0.05, ** *p*  <  0.01, *** *p*  <  0.001, and **** *p*  <  0.0001.

Co-treatment with HB and SD2 reduced the MG-63 cell viability across all tested combinations and time points (24 h and 72 h) (Figure 5a and Figure 6a). However, the combinations that contained 80% of the HB IC_50_ with 20% of the SD2 IC_50_, as well as 60% HB with 40% SD2, showed weaker effects compared with HB alone. The combination of 80% HB + 20% SD2 was also the only condition that did not result in statistically significant reduction in the viability. In contrast, the 20% HB + 80% SD2 combination significantly reduced the cell viability relative to the control (*p* < 0.01), although its effect was less pronounced than that of SD2 alone, suggesting a potentiated effect of SD2. Notably, this combination was more effective than the 80% HB + 20% SD2 treatment. The cytotoxic effects were more pronounced at 72 h, where almost all combinations significantly inhibited the cell viability at this time point. In the Saos-2 cells, the cytotoxic effects at 24 h were generally comparable across the treatment groups (Figure 5b and Figure 6b). The 80% HB + 20% SD2, 50% HB + 50% SD2, and 40% HB + 60% SD2 combinations exhibited the weakest cytotoxicities. Conversely, their reciprocal combinations—60% HB + 40% SD2 and 20% HB + 80% SD2—resulted in significant reductions in the cell viability. By 72 h, all treatment combinations led to significant cytotoxic effects in the Saos-2 cells. In the hFOB 1.19 cells, all treatment combinations except 80% HB + 20% SD2 significantly reduced the cell viability at 24 h (Figure 6c). The combinations with a higher proportion of SD2 than HB produced stronger cytotoxic responses (Figure 5c). At 72 h, a partial recovery of cell viability was observed, with the most notable recovery in the 80% HB + 20% SD2 group. Similarly, the cells treated with HB at its exact IC_50_ concentration also showed signs of recovery over time. These results suggest a time-dependent response in the hFOB 1.19 cells, with reduced long-term cytotoxicity under certain treatment conditions.

The effects of the HB and SD2 co-treatment on reactive oxygen species (ROS) production varied between the three cell lines. In the MG-63 cells, the combined treatments did not elicit significant oxidative stress (Figure 6d). In several cases, the ROS levels were lower than those observed in the untreated controls, suggesting a potential protective or antioxidative effect of certain HB–SD2 combinations. In contrast, the Saos-2 cells displayed markedly elevated ROS production following the co-treatment, particularly in combinations with higher proportions of SD2 (Figure 6e). The treatments with SD2 alone (*p*  <  0.05), 20% HB + 80% SD2 (*p*  <  0.01), and combined IC_50_ concentrations (*p*  <  0.01) resulted in statistically significant increases in the ROS levels compared with the control, indicating a more pronounced oxidative response in this cell line. In the hFOB 1.19 cell line, the ROS levels remained relatively low across most treatment groups (Figure 6f). Nevertheless, some combinations with a higher SD2 content led to a statistically significant increase in the ROS production relative to the untreated cells.

### 2.3. Apoptotic and Necrotic Responses of HB and SD2 Co-Treatment Through Flow Cytometry Analysis

The effects of HB and SD2 on cell death were comprehensively analyzed in terms of the apoptotic and necrotic responses using flow cytometry following the treatments at varying doses and two time points.

**Figure 7:**
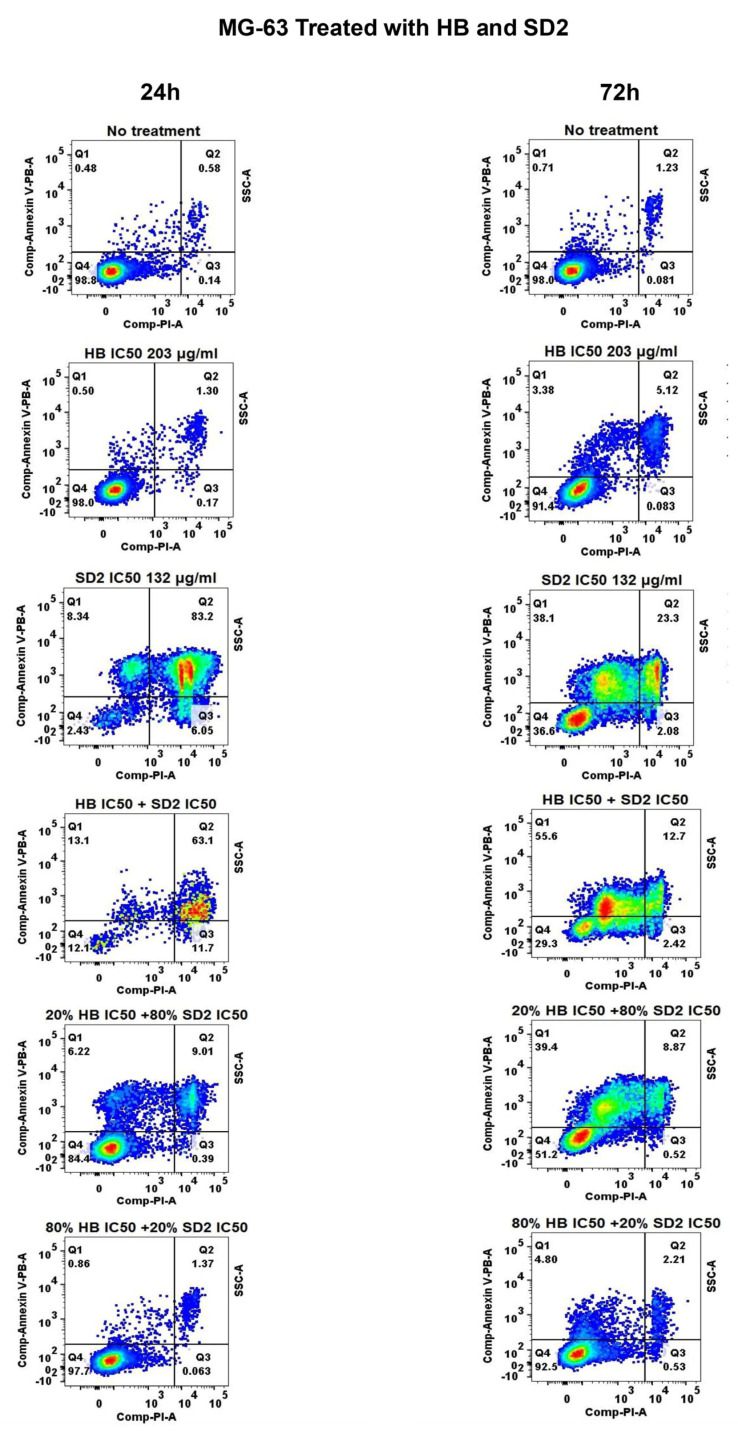
Effects of HB, SD2, or a combination of HB + SD2 on the apoptosis profiles of MG-63 cell lines. The no treatment panel corresponds to the untreated cells used as the negative control. The treatment panels correspond to 24 h and 72 h treatments with HB, SD2 or combination. Cell apoptosis was measured using Annexin V-PB/PI double staining. The results are expressed as the percentage of cells that corresponded to Q1: early apoptotic cells (Annexin V+/PI−), Q2: late apoptotic cells (Annexin V+/PI+), Q3: necrotic cells (Annexin V− PI+), and Q4: healthy cells (Annexin V−/PI−).

**Figure 8:**
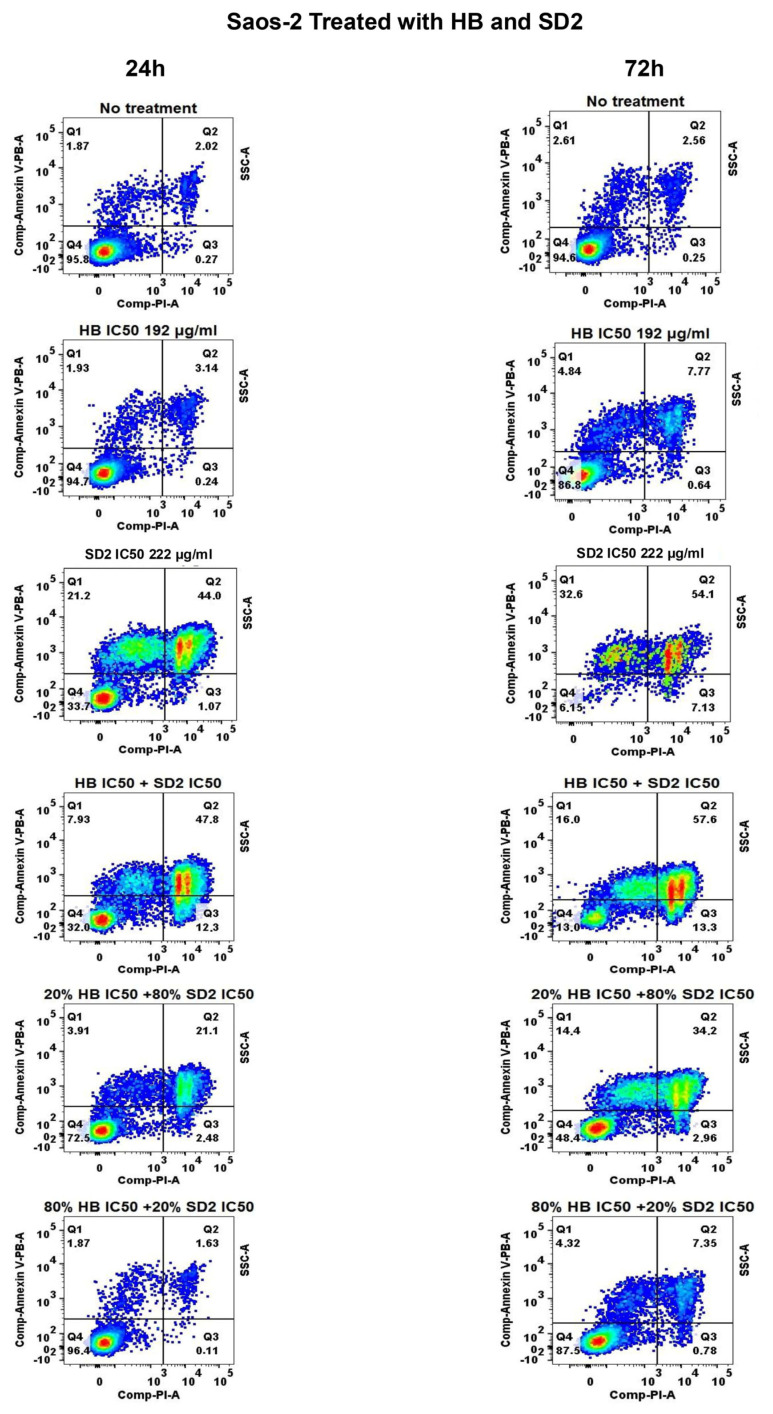
Effects of HB, SD2, or a combination of HB + SD2 on the apoptosis profiles of Saos-2 cell line. The no treatment panel corresponds to the untreated cells used as the negative control. The treatment panels correspond to the 24 h 72 h treatments with HB, SD2 or combination. Cell apoptosis was measured using Annexin V-PB/PI double staining. The results are expressed as the percentage of cells corresponding to Q1: early apoptotic cells (Annexin V+/PI−), Q2: late apoptotic cells (Annexin V+/PI+), Q3: necrotic cells (Annexin V− PI+), and Q4: healthy. Cells (Annexin V−/PI−).

**Figure 9:**
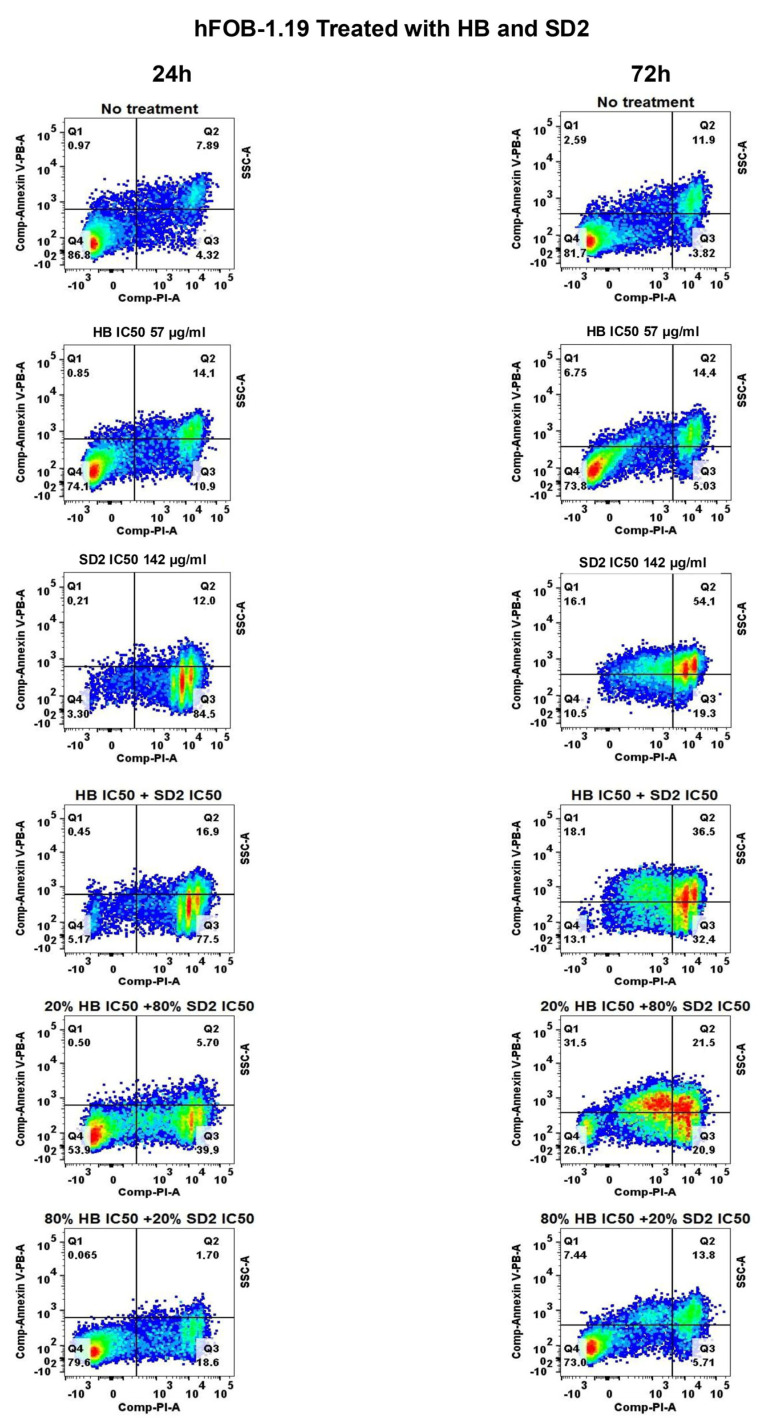
Effects of HB, SD2, or a combination of HB + SD2 on the apoptosis profiles of hFOB 1.19 cell line. The no treatment panel corresponds to the untreated cells used as the negative control. The treatment panels correspond to the 24 h and 72 h treatment with HB, SD2 or combination. Cell apoptosis was measured using Annexin V-PB/PI double staining. The results are expressed as the percentage of cells corresponding to Q1: early apoptotic cells (Annexin V+/PI−), Q2: late apoptotic cells (Annexin V+/PI+), Q3: necrotic cells (Annexin V− PI+), and Q4: healthy. Cells (Annexin V−/PI−).

Apoptotic profiles of the cell lines after 24 h and 72 h of treatment with HB, SD2 (at their respective IC_50_ concentrations), and their combination, alongside the profiles of untreated cells, are shown in Figure 7, Figure 8, and Figure 9.

HB did not exhibit any apoptotic effect on the MG-63 cells after 24 h, while the SD2 treatment exerted a significant effect characterized by a decrease in healthy cells and an increase in the early and late apoptotic cells (Figure 7). In the Saos-2 cells, the treatment with HB at its IC_50_ concentration also did not cause a significant apoptotic effect after 24 h (Figure 8), and SD2 at its IC_50_ concentration had a significantly more potent effect, which sharply reduced the healthy cell percentage to 62.50%, which was accompanied by an increase in the early apoptosis to 12.80%, and a dramatic surge in the late apoptotic cells to 39.98%. Furthermore, a substantial proportion of cells (17.77%) were found to be necrotic. The treatment of the hFOB 1.19 cells with HB at its IC_50_ concentration reduced the healthy cell percentage to 74.35% after 24 h (Figure 9). This was accompanied by a weak increase in early apoptotic cells (4.97% ± 0.05%), late apoptotic cells (13.43%), and necrotic cells (9.75%). SD2 at its IC_50_ concentration had a more substantial impact on the hFOB 1.19 cells, which decreased the healthy cell proportion to 46.48% and was accompanied by increases in the early apoptotic (9.62%), late apoptotic (27.98%), and necrotic (15.93%) levels.

The HB treatment of MG-63 after 72 h resulted in minimal apoptotic activity (Figure 7). SD2 slightly reduced the proportion of healthy cells, with corresponding increases in the early (5.24%) and late apoptosis (6.68%). The similarity between the 24 and 72 h profiles suggests that prolonged SD2 exposure did not enhance the apoptotic induction in the MG-63 cells, consistent with the cytotoxicity data. In Saos-2 cells, the HB treatment for 72 h maintained a high proportion of healthy cells (Figure 8), although a slight increase in late apoptotic cells (8.23%) was noted compared with the results acquired at 24 h. In contrast, SD2 reduced the proportion of viable cells to 45.64%, where the late apoptotic cells reached 30.38%—a value nearly identical to that at 24 h. The treatment with HB moderately reduced the proportion of healthy hFOB 1.19 cells to 72.20% after 72 h (Figure 9), whereas SD2 led to a more pronounced decline to 46.42%, which was accompanied by increases in both the apoptotic and necrotic populations. Notably, the apoptotic distribution remained largely consistent between the 24 and 72 h time points.

The apoptotic profiles of the MG-63 cell line after 24 and 72 h of treatment with various combinations of HB and SD2 showed distinct cellular responses (Figure 7, Supplementary Figure S2). The combination of 20% HB + 80% SD2 exerted the strongest effect, where it reduced the proportion of healthy cells to 79.08% and slightly increased the percentages of early and late apoptotic cells to 4% and 6%, respectively, after 24 h of treatment. Conversely, the treatment with 80% HB + 20% SD2 largely preserved the cell viability, suggesting that a higher proportion of HB may have mitigated the cytotoxic effects of SD2 in the MG-63 cells. After 72 h of treatment, the combination of HB IC_50_ and SD2 IC_50_ continued to demonstrate a strong synergistic effect in the MG-63 cells, which reduced the proportion of healthy cells to 22.63%. This was accompanied by a substantial increase in the early apoptotic cells (59.73%), while late apoptosis and necrosis remained relatively low at 7.01% and 1.12%, respectively. This shift toward a predominant early apoptotic profile suggests a more defined apoptotic commitment over time. The evaluation of combination ratios revealed dose-dependent modulation. The combination 20% HB + 80% SD2 showed moderate cytotoxicity, where it reduced the percentage of healthy cells to 47.85%, and 29.95% underwent early apoptosis and 6.46% underwent late apoptosis. Necrosis remained minimal at 0.66%. In contrast, the 80% HB + 20% SD2 combination preserved the cell viability at 92.43%, indicating a marked reduction in cytotoxicity compared with the equimolar or SD2-dominant combinations. Collectively, these findings reinforce the time- and ratio-dependent natures of the HB–SD2 co-treatment and suggest that a higher proportion of SD2 drives apoptotic activity, particularly in the early stages, while HB may attenuate this effect when present in excess.

The impact of the combined treatment of HB and SD2 on the apoptotic profiles of the Saos-2 cell line after 24 and 72 h is shown in Figure 8 (and Supplementary Figure S3). The different combinations of HB with SD2 presented the significant apoptotic effect in a SD2-dose-dependent manner on the Saos-2 cell lines after 24 h. The cotreatment of HB and SD2 at IC_50_ concentrations demonstrated a profound impact, which led to a further reduction in the Saos-2 cell healthy profile to 32.95%. The percentage of apoptotic cell subpopulations were measured as 13.96%, 48.45%, and 6.87% in the early, late, and necrotic phases, respectively. The 20% HB + 80% SD2 combinatory treatment decreased the proportion of healthy cells to 79.23% while increasing the early apoptotic cells percentage to 13.76%, late apoptotic cells to 33.44%, and necrotic cells to 4.78%. Once again, with the dominant proportion of HB, the 80% HB + 20% SD2 combination largely preserved cell viability. Similar to the MG-63 cells, a higher proportion of HB in the combination appeared to reduce the cytotoxic effects observed with the SD2-dominant treatments in the Saos-2 cells. At 72 h, the co-treatment of HB and SD2 continued to exert a significant apoptotic effect on the Saos-2 cells in a SD2-dose-dependent manner. The combination of HB IC_50_ and SD2 IC_50_ led to a dramatic decrease in the healthy cell population to 3.14%, accompanied by a pronounced increase in early apoptotic cells (41.95%) and late apoptotic cells (51.20%). Necrosis was also observed at 8.45%. The 20% HB + 80% SD2 combination maintained a moderate apoptotic response, which reduced the healthy cells to 52.35%, while increasing the early and late apoptotic populations to 8.09% and 24.23%, respectively, along with 22.28% necrosis. Consistent with previous observations, the 80% HB + 20% SD2 combination largely preserved the cell viability at 88.43%, with minimal apoptotic and necrotic activity. These results reinforce the trend observed at 24 h, where increasing the proportion of SD2 in the co-treatment enhanced the cytotoxic and apoptotic responses, while a dominant presence of HB appeared to attenuate these effects in the Saos-2 cells.

The apoptotic profiles of the hFOB 1.19 cell line after 24 and 72 h of treatment with various combinations of HB and SD2 are shown in Figure 9 and Supplementary Figure S4. The combination of HB IC_50_ and SD2 IC_50_ after 24 h of treatment demonstrated the most pronounced effect on the hFOB 1.19 cells, which resulted in a dramatic reduction in healthy cells to 18.09%, while the percentage of necrotic cells surged to 46.80% without a significant change in the apoptotic subpopulations, suggesting that this combination might have induced a significant level of cell death through necrosis in the hFOB 1.19 cells. Further analysis of the combination ratios showed that 20% HB + 80% SD2 led to 40.83% healthy cells, which was associated with elevated early apoptosis (8.43%), late apoptosis (21.95%), and necrosis (25.83%) proportions. In contrast, the 80% HB + 20% SD2 combination allowed 80% of the cells to remain healthy, with a negligible effect on the apoptotic components. Similar to the other cell lines, a higher proportion of HB in the combination generally mitigated the more severe cytotoxic effects observed with higher SD2 concentrations. At 72 h, the combination of HB IC_50_ and SD2 IC_50_ continued to exert a pronounced effect on the hFOB 1.19 cells, where it further reduced the proportion of healthy cells to approximately 17.5% and drove a notable increase in necrotic cells to around 30.0%, while the apoptotic subpopulations remained relatively unchanged. After the 20% HB + 80% SD2 treatment, the healthy cell percentages dropped to about 25.0%, accompanied by elevations in early apoptosis (approximately 15.0%) and late apoptosis (around 26.0%), with a moderate necrotic fraction observed (~28.0%). Conversely, the 80% HB + 20% SD2 combination largely preserved the cell viability, where 78% of the cells remained healthy, with only minimal changes in the apoptotic and necrotic components. Overall, as observed at 24 h, a higher proportion of HB in the combination mitigated the severe cytotoxic effects associated with SD2-dominant treatments, indicating that HB may have exerted a protective influence on the hFOB 1.19 cells, even after extended exposure.

The flow cytometry analysis revealed that the SD2 and HB agents elicited distinct biological responses in the healthy and cancerous cell lines. In the normal osteoblast cell line hFOB 1.19, the SD2 treatment led to a dose-dependent increase in apoptosis and necrosis, with marked cellular stress and toxicity observed at higher concentrations. In contrast, HB exhibited a milder cytotoxic effect on the hFOB 1.19 cells, with a higher proportion of viable cells retained. In the osteosarcoma cell lines MG-63 and Saos-2, SD2 caused a significant reduction in the viable cell populations and induced a strong pro-apoptotic effect, as evidenced by increased early and late apoptosis. While HB also triggered apoptosis in these cancer cells, its effect was less pronounced compared with SD2. Under combined treatment conditions, co-administration of SD2 and HB led to further increases in the apoptotic cell populations relative to single-agent treatments. This synergistic effect was particularly evident in the late apoptosis rates and suggested an enhanced therapeutic efficacy. In conclusion, the combination of SD2 and HB potentiated apoptosis in the cancer cells while eliciting a more controlled response in the non-tumor cells, which supported the selective antitumor potential of SD2.

### 2.4. Evaluation of the HB and SD2 Treatment Effects on the 3D Cell Cultures

**Figure 10:**
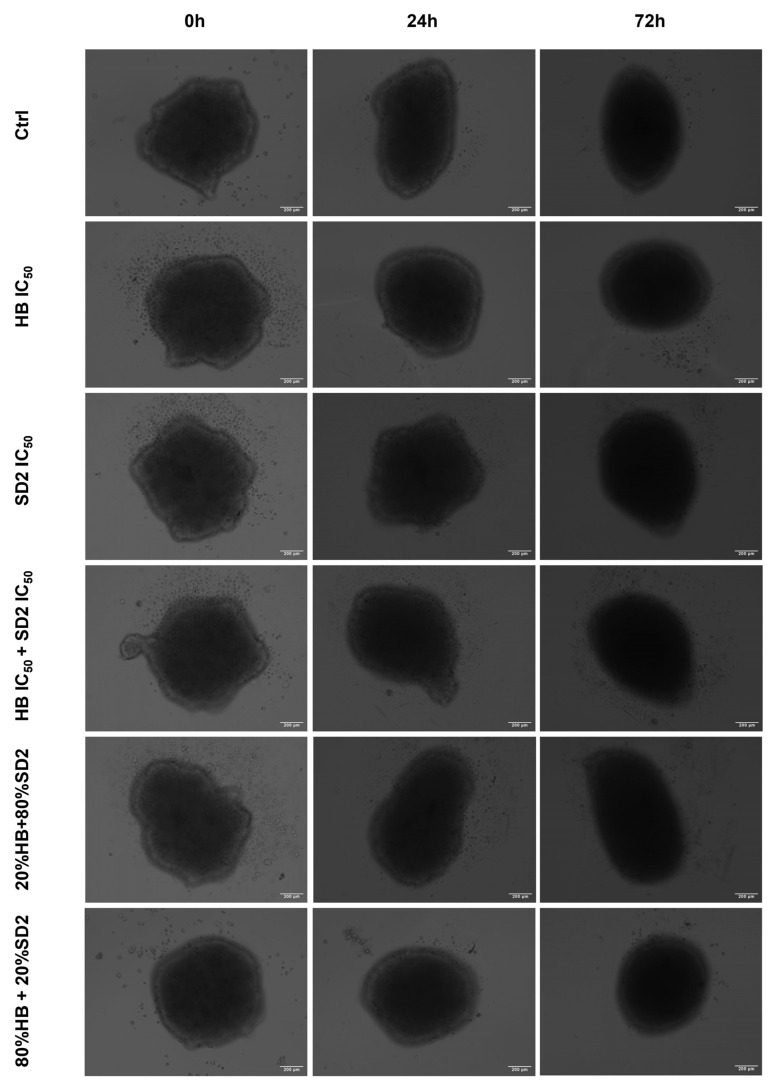
Representative transmitted light microscopy images of the MG-63 spheroids incubated with combined treatments of halogenated boroxine (HB) and Dex-CeNPs (SD2) for 24 h or 72 h, as indicated. The scale bar indicates a size of 200 µm.

**Figure 11:**
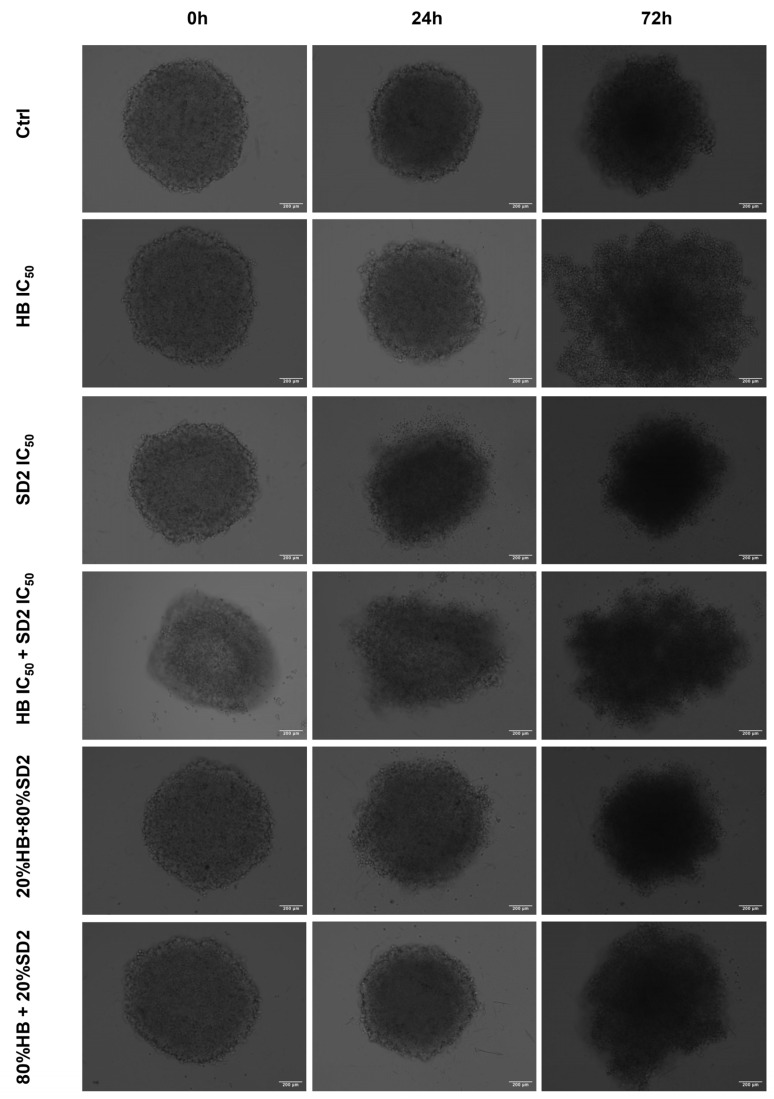
Representative transmitted light microscopy images of the Saos-2 spheroids incubated with combined treatments of halogenated boroxine (HB) and Dex-CeNPs (SD2) for 24 h or 72 h, as indicated. The scale bar indicates a size of 200 µm.

**Figure 12:**
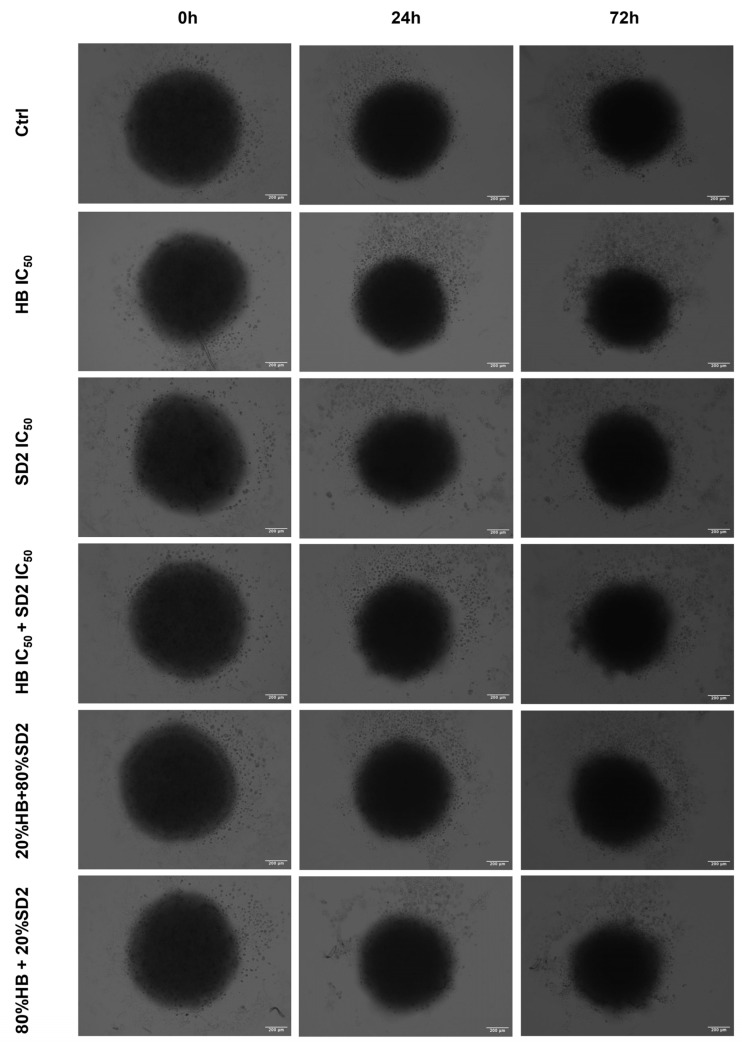
Representative transmitted light microscopy images of the hFOB 1.19 spheroids incubated with combined treatments of halogenated boroxine (HB) and Dex-CeNPs (SD2) for 24 h or 72 h, as indicated. The scale bar indicates a size of 200 µm.

**Figure 13:**
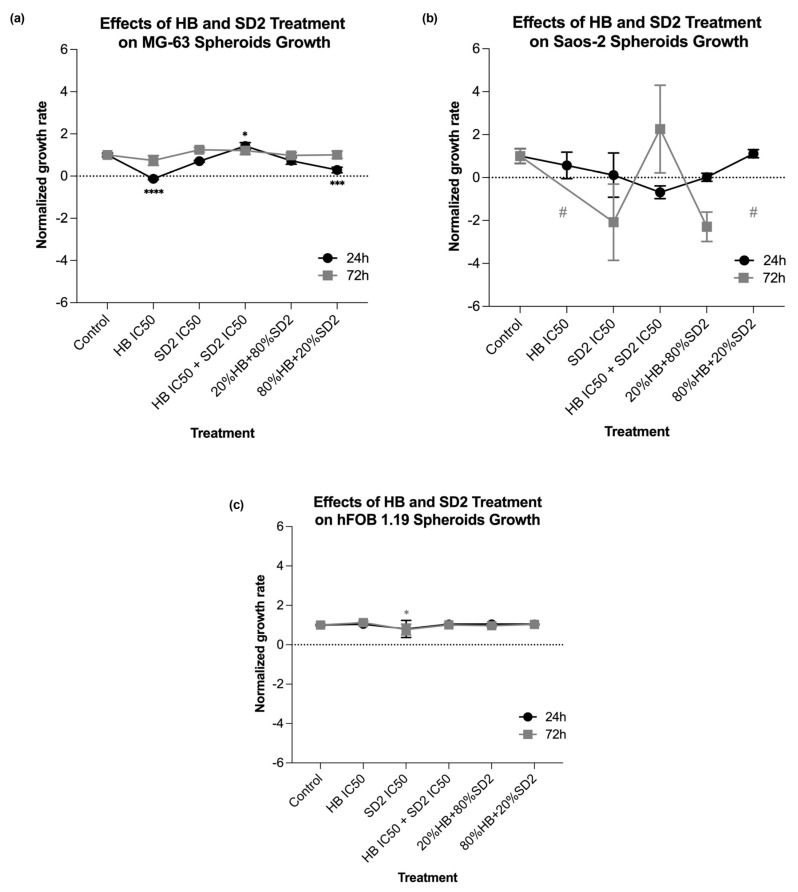
Effects of halogenated boroxine (HB) and Dex-CeNPs (SD2) co-treatments on the growth rates of MG-63 (**a**), Saos-2 (**b**), and hFOB 1.19 (**c**) spheroids. The Saos-2 spheroids treated with HB IC50 and 80% HB + 20% SD2 were disrupted at 72 h and could not be measured (marked with #). Statistical significance is indicated as * *p*  <  0.05, *** *p*  <  0.001, and **** *p*  <  0.0001.

All three cell lines were successfully cultivated in 3D cultures, with observable changes over the time of the exposure (Figure 10, Figure 11 and Figure 12). These changes were more pronounced in the cancer cell lines, especially in the Saos-2 spheroids, where certain treatments—namely, HB alone at its IC_50_ concentration and the combination of 80% HB + 20% SD2—caused disruptions that made measurement impossible after 72 h (Figure 11 and Figure 13b).

When evaluating the effects of the tested treatments on the spheroids, the growth rates of both osteosarcoma spheroid types were affected. In the MG-63 spheroids (Figure 13a), the treatment with HB alone and the combination of 80% HB + 20% SD2 significantly reduced its growth rate at 24 h; however, the growth appeared to recover after 72 h. Interestingly, the treatment with the combination of IC_50_ values of both compounds resulted in a slight but statistically significant increase in the growth rate compared with the control at 24 h (*p*-value = 0.0369), while a decrease was observed at 72 h. In the Saos-2 spheroids, no treatment showed a statistically significant effect compared with the control (Figure 13b). Nevertheless, based on the normalized growth rate profiles and morphological observations, the Saos-2 spheroids appeared to be the most affected. The treatment with the combination of HB IC_50_ and SD2 IC_50_ decreased the normalized growth rate after 24 h, but an increase was observed at 72 h. On the other hand, SD2 alone and the combination of 20% HB + 80% SD2 led to a time-dependent decrease in the normalized growth rate of the Saos-2 spheroids. Markedly, the hFOB 1.19 spheroids were the least affected. There were no substantial differences between the treated and control spheroids, nor were any time-dependent changes observed (Figure 13c).

## 3. Discussion

Using various experimental approaches, the results of this study integrate data on the effects of HB, SD2, and their co-treatments in tumorigenic (MG-63 and Saos-2) and non-tumor (hFOB 1.19) cell lines. Observations from individual and combined treatments are contextualized in the following section across multiple levels of analysis—starting with the cytotoxicity and ROS profiles, followed by the apoptotic and necrotic responses, and concluding with the 3D spheroid growth dynamics.

Our research showed that HB exerted dose- and time-dependent cytotoxic effects on both the MG-63 and Saos-2 osteosarcoma cell lines, which is in line with previous studies that established its pro-apoptotic and cytotoxic activities in hematological and solid malignancies [19,20,21]. In particular, the observed reduction in cell viability at higher HB concentrations corroborates findings in UT-7 leukemia cells, where HB triggered apoptosis via caspase activation and the deregulation of pro-survival genes [19,20]. Similarly, Elez-Burnjakovic et al. reported HB-induced cytotoxicity and the modulation of autophagy in melanoma and bladder carcinoma cells, implicating oxidative stress and metabolic reprogramming as possible underlying mechanisms [21,22].

Interestingly, the lowest concentration of HB (50 µg/mL) resulted in a slight increase in viability at 72 h in the MG-63 cells, suggesting a possible low-dose-induced adaptive response or mild cellular stimulation. This dual effect has been described in other cancer models exposed to boron-based agents, where low doses may activate compensatory survival pathways [30]. In contrast, the hFOB 1.19 cells exhibited a higher sensitivity to HB, with cytotoxicity apparent even at moderate concentrations. However, the response in this non-tumor line lacked a strict dose-dependent trend, indicating specific responses to HB, which may involve differences in the oxidative resilience, baseline metabolic activity, or other background mechanisms, which should be examined at the molecular level.

The cytotoxicity assessment of SD2 also revealed concentration- and cell-line-dependent responses across the osteosarcoma (MG-63 and Saos-2) and non-tumor osteoblastic (hFOB 1.19) cell models. Notably, the MG-63 cells demonstrated a biphasic, hormetic response, wherein a low concentration of SD2 (100 µg/mL) elicited a modest increase in viability at 24 h, suggesting the potential transient activation of survival pathways, followed by a pronounced reduction in viability at concentrations ≥ 250 µg/mL. This phenomenon is consistent with the hormetic behavior previously described for cerium oxide nanoparticles, wherein low-dose exposure activates cytoprotective antioxidant pathways, while higher concentrations shift the redox balance via Ce^3+^/Ce^4+^ cycling, generating reactive oxygen species (ROS) that lead to mitochondrial dysfunction and apoptosis [31,32].

In contrast, the Saos-2 cells exhibited a relatively chemoresistant phenotype, where they showed less severe viability reduction and partial recovery over 72 h. This attenuated response likely reflects cell-line-specific differences in basal oxidative status, nanoparticle uptake, and redox buffering capacity [33]. Interestingly, the hFOB 1.19 line displayed the steepest early viability reduction (20–35% at 500–1000 µg/mL, 24 h), yet recovered to 60–70% by 72 h. Although the ROS levels were already suppressed below the control at all doses, the transient viability loss may stem from non-oxidative stresses, such as lysosomal overload or ionic imbalance. The rapid rebound concurs with SD2’s catalase/SOD-mimetic activity that continually scavenges ROS in normal osteoblasts, allowing metabolic adaptation once the acute nanoparticle burden is resolved [34,35,36]. Mechanistically, the redox-switching property of cerium oxide is central to SD2’s biological effects. At lower concentrations, cerium oxide nanoparticles scavenge free radicals and reduce oxidative stress, whereas higher doses promote oxidative damage through ROS accumulation, triggering apoptosis via mitochondrial and caspase-mediated pathways [35,36]. The dextran coating of SD2 significantly influences these interactions, possibly by enhancing the colloidal stability, facilitating endocytosis, and modulating the nanoparticle’s intracellular fate through its specific branching configuration, which could alter the protein corona and cellular trafficking dynamics [37]. This may explain the steeper cytotoxic thresholds observed with SD2 compared with other surface coatings, such as polyethylene glycol (PEG) or polyvinylpyrrolidone (PVP), which have been shown to mitigate cerium-oxide-nanoparticle-induced toxicity [38].

Moreover, the observed intercellular heterogeneity in response to SD2 underscores the importance of considering tumor-specific oxidative metabolism and nanoparticle processing mechanisms. The heightened sensitivity of the MG-63 cells may reflect an increased nanoparticle uptake and a less competent antioxidant defense system, while the resilience of the hFOB 1.19 cells suggests efficient metabolic adaptation and the possible exploitation of cerium oxide nanoparticles’ catalytic scavenging over time [39].

Overall, SD2 elicited a concentration-dependent cytotoxic response that was most pronounced in the MG-63 osteosarcoma cells, displayed moderate effects in the Saos-2, and caused only transient impairment in the non-tumor hFOB 1.19 cells. These findings, which are consistent with the redox-switching behavior of dextran-coated cerium oxide nanoparticles reported in the literature, underscore SD2’s potential as a selective nanotherapeutic for osteosarcoma, while highlighting the necessity for dose optimization and mechanistic validation.

The reactive oxygen species (ROS) generation profiles revealed that high HB concentrations significantly increased the oxidative stress in osteosarcoma cells. This supports the hypothesis that HB’s cytotoxicity is mediated, at least in part, by oxidative mechanisms, as previously demonstrated in UT-7 and GR-M cells, where HB altered the expression of redox- and apoptosis-related genes [19,20,21]. Conversely, low HB concentrations reduced ROS production, suggesting a threshold effect or ROS scavenging at sub-cytotoxic levels. In the hFOB 1.19 cells, the ROS induction was modest and not statistically significant across most conditions, which possibly reflected stronger antioxidant defenses in non-malignant osteoblasts.

SD2 caused markedly divergent reactive oxygen species (ROS) responses that tracked both the dose and cellular context. In the MG-63 osteosarcoma cells, the intracellular ROS levels remained near the baseline at 100–250 µg/mL but surged to nearly three-fold above the control levels at 500–1000 µg/mL, which mirrored the steep viability decline observed at these concentrations. This response aligns with previous findings where cerium oxide nanoparticles induced oxidative-stress-mediated cytotoxicity through mitochondrial ROS production and apoptosis [35,40].

The Saos-2 cells exhibited an even more pronounced pro-oxidant profile, with significant ROS elevation already detectable at 100 µg/mL and a plateau observed around 250 µg/mL, suggesting earlier saturation of nanoparticle-induced oxidative stress [39]. These differences likely stemmed from cell-line-specific variations in the basal redox state, nanoparticle internalization kinetics, and antioxidant capacity [33,34].

Strikingly, the hFOB 1.19 displayed a consistent reduction in ROS levels across all SD2 concentrations, with the DCFDA signals falling to nearly 30–40% of the control at 250–1000 µg/mL. This observation supports the proposed role of cerium oxide nanoparticles as potent ROS scavengers in normal cells, likely via catalase- and superoxide dismutase (SOD)-mimetic mechanisms that become prominent under physiological pH and normoxic conditions [32,36]. Collectively, these findings underscore SD2’s unique redox-modulatory properties, which are pro-oxidative in malignant osteosarcoma cells and antioxidative in non-transformed osteoblastic cells, thus supporting its potential utility as a context-sensitive nanotherapeutic agent in bone oncology [24,29].

The co-treatment with HB and SD2 produced complex, concentration-ratio-dependent effects on the cell viability and oxidative stress. In the MG-63 and Saos-2 cells, most HB–SD2 combinations induced cytotoxicity, though some combinations (notably 80% HB + 20% SD2 and 60% HB + 40% SD2) were less effective than HB alone. These findings suggest potential antagonistic interactions at certain ratios, possibly due to the ROS-scavenging properties of cerium oxide nanoparticles (CeNPs), which can neutralize the oxidative effects of HB [29]. In support of this, combined treatments in the MG-63 cells did not increase the ROS production, and in some cases, the ROS levels were lower than in untreated controls. These observations aligned with studies that have shown the dual redox nature of CeNPs, which can act as pro-oxidants in tumor microenvironments while protecting non-malignant cells from oxidative damage [24,29].

The Saos-2 cells responded to the co-treatment with elevated ROS levels, particularly for combinations rich in SD2. The increase in oxidative stress suggests that the balance between SD2’s redox activity and the intrinsic oxidative state of the Saos-2 cells may differ from that in the MG-63 cells, potentially reflecting metabolic or mitochondrial differences between the lines. Furthermore, the pronounced ROS response in the Saos-2 cells following the 20% HB + 80% SD2 treatment supports the notion that CeNPs can shift from antioxidative to oxidative behavior depending on the cellular context and nanoparticle dose [29].

The behavior of the hFOB 1.19 cells under co-treatment conditions was notably different. While most combinations reduced the viability at 24 h, partial recovery was observed at 72 h. This suggests that the hFOB 1.19 cells may have engaged repair or adaptation mechanisms in response to early oxidative or cytotoxic stress, particularly when HB was present at lower ratios. Notably, the HB treatment at its IC_50_ concentrations alone also showed signs of reduced cytotoxicity over time in the hFOB 1.19 cells, indicating potential cellular resilience or activation of detoxification pathways that mitigate long-term damage. This recovery effect was not observed in the HB screening experiments; however, in those experiments, as mentioned before, the cells did not show a linear response to HB’s cytotoxic effect, implying that healthy osteoblasts show concentration-specific and potentially transient cellular responses, possibly representing a narrow threshold at which normal cells can engage protective mechanisms.

Our findings in the 3D cell cultures revealed differential spheroid responses to HB, SD2, and their combinations across the tested cell lines. In the MG-63 spheroids, both HB alone and the 80% HB + 20% SD2 combination significantly reduced the growth at 24 h, followed by signs of recovery by 72 h. Notably, the IC_50_ combination paradoxically increased the growth at 24 h, which may indicate sub-lethal stress-induced proliferation or altered spheroid compactness, warranting further mechanistic investigation. Although the Saos-2 spheroid growth did not show statistically significant differences, the morphological indicators and normalized growth profiles suggested greater sensitivity to treatment, particularly with SD2 alone and the 20% HB + 80% SD2 combination. These treatments elicited a time-dependent decrease in the spheroid growth. The hFOB 1.19 spheroids demonstrated a remarkable resistance to all the treatments, with no significant changes in growth or morphology. This highlights and reconfirmed their relative robustness and corroborated the selective cytotoxicity of HB and SD2 toward malignant cells. Although the effects of HB in 3D culture systems have not yet been explored, its selective cytotoxicity has been extensively documented in 2D models [18,19,20,21,22], and our findings in spheroids appear to corroborate this selectivity. Overall, our data emphasize the importance of evaluating spheroid dynamics over multiple time points and integrating both quantitative and qualitative endpoints to comprehensively assess the therapeutic efficacy in 3D models.

## 4. Materials and Methods

### 4.1. Synthesis and Preparation of Halogenated Boroxine K_2_(B_3_O_3_F_4_OH) (HB) and Dextran-Coated Cerium Oxide Nanoparticles (Dex-CeNPs)

HB is a white powder that is soluble in water (7.1% at 20 °C), ethanol, and DMSO. It was synthesized according to a previously described protocol with the slight modification [41]. The stock solutions were prepared by dissolving 20 mg of K_2_(B_3_O_3_F_4_OH) in 1 mL of 1× PBS, and the final concentrations of the solutions to be tested were made in the cell culture medium.

The synthesis of dextran-coated cerium oxide nanoparticles (Dex-CeNPs), here labeled as SD2, was carried out according to the protocol published by Tarakci et al., 2025 [29]. In that study, the protocol was well optimized, and a detailed physicochemical characterization was conducted. Based on this, in the present study, cerium oxide nanoparticles coated with dextran from *Leuconostoc* spp. (Sigma, Cat# 31388, mol wt: ~6 kDa) (SD2) were used [29]. For each newly synthesized SD2 sample, basic characterization was once again performed, the concentration was determined, and solutions of a defined concentration were prepared in the cell culture medium for testing (Supplementary Figure S1).

### 4.2. Cell Lines and Cell Culture Maintenance

Two osteosarcoma cell lines—MG-63 (CRL-1427) and Saos-2 (HTB-85)—and one osteoblast cell line—hFOB 1.19 (CRL-3602)—were obtained from the American Type Culture Collection (ATCC, Manassas, VA, USA). The MG-63 cells were cultured in Eagle’s Minimum Essential Medium (Sigma-Aldrich, Saint Louis, MO, USA), the Saos-2 cells were cultured in McCoy’s 5a Medium Modified (Sigma-Aldrich), and the hFOB 1.19 cells were maintained in Dulbecco’s Modified Eagle’s Medium/Nutrient Mixture F-12 Ham (Sigma-Aldrich). All cell media were supplemented with 10% fetal bovine serum (FBS, Belize City, Belize) and 1% penicillin–streptomycin, and were incubated in a humidified atmosphere of 5% CO_2_ in air and at a temperature of 37 °C. All were also tested on mycoplasma periodically using MycoAlert test (Lonza, Walkersville, MD, USA). The cell line identity was confirmed using PowerPlex^®^ Fusion System (Promega, Madison, WI, USA) and STR profiles available in the ATCC database.

### 4.3. HB Treatments

The experiments were carried out in 96-well microtiter plates, with 5000 cells seeded per well. After 24 h, the cell cultures were treated with HB at final concentrations of 50, 75, 100, 150, 200, and 400 µg/mL. Due to the different sensitivity, the hFOB 1.19 cells were additionally treated with HB at a final concentration of 25 µL/mL. Each treatment was carried out in triplicate and experiments were repeated at least twice. Untreated cells were used as controls. The cell viabilities of the MG-63, Saos-2, and hFOB 1.19 cells cultured in the presence of HB were calculated as a percentage of the control cells, and the half-maximal inhibitory concentration (IC_50_) values were obtained from the dose–response curves plotted for the results obtained after 24 h.

### 4.4. SD2 Treatments

To evaluate the effects of SD2, the MG-63, Saos-2, and hFOB 1.19 cells were also seeded at a density of 5000 cells per well in 96-well plates. Following a 24 h incubation period, the cells were exposed to SD2 at concentrations of 100, 250, 500, and 1000 µg/mL. All treatments were performed in triplicate and independently repeated at least three times to ensure reproducibility. Untreated cells served as negative controls. The cell viability was assessed relative to the control group, as in the HB screening experiments, and the dose–response curves were generated to determine the IC_50_ values for each cell line 24 h post-treatment.

### 4.5. HB and SD2 Co-Treatments

The IC_50_ values obtained from the single-drug cell viability/cytotoxicity assays were used to design subsequent drug combination experiments for each particular cell type. The following combined/synergistic treatment ratios were defined: 0.8-, 0.6-, 0.5-, 0.4-, and 0.2-fold of the HB IC_50_ with 0.2-, 0.4-, 0.5-, 0.6-, and 0.8-fold of the SD2 IC_50_, respectively. In addition, a combination of both compounds’ IC_50_ concentrations was tested. All treatments were performed in triplicate, with at least three independent experimental replicates.

### 4.6. Cytotoxicity Assay

The cytotoxic activity was determined using the WST-1 assay and it was performed at two time points: at day 1 (24 h after treatment) and day 3 (72 h after treatment). Upon the incubation of 24 h and 72 h after the treatments of cells, the medium containing the treatments was removed from each well, and 100 μL of a WST-1 reagent (1:10 ratio with cell culture medium) was added to each well and incubated for 2 h. At the end of the incubation, a color change from pink to light brown was observed, and the absorbances of each well were measured at 450 nm and 650 nm using a BioTek Cytation 5 Cell imaging multimode reader. The viability/cytotoxicity percentages were counted, and graphs were plotted by normalizing the data against the untreated control groups.

### 4.7. Reactive Oxygen Species (ROS) Detection

To assess the reactive oxygen species (ROS), a 2′, 7′- dichlorofluorescein diacetate (DCFDA) assay was used. The ROS levels were measured simultaneously with each cytotoxicity 24 h post-treatment, with the same treatments and controls (untreated cells) applied.

After the end of the incubation period of 24 h, the medium with treatments was removed, and each well was washed twice with 1× PBS. The remaining cells were then incubated with 10 µM DCFDA in the dark for 45 min at 37 °C in a 5% CO_2_. Following the incubation, the fluorescence intensity was measured in the 485 nm/535 nm range using a BioTek Cytation 5 Cell imaging multi-mode reader. The ROS-related fluorescence intensity fold changes were calculated and normalized based on the cell viability data.

### 4.8. Flow Cytometry Analysis

The flow cytometry analysis was performed to evaluate the cell viability using the APC Annexin V Apoptosis Detection Kit with PI (BioLegend) specifically designed for the identification of healthy, apoptotic, and necrotic cells. Based on the staining patterns, the cells were classified as follows: healthy—Annexin-V^−^/PI^−^, early apoptotic—Annexin-V^+^/PI^−^, late apoptotic—Annexin-V^+^/PI^+^, and necrotic—Annexin-V^−^/PI^+^.

To perform the flow cytometry, 300,000 cells per well were seeded in a 6-well plate and incubated for 24 h. Following the incubation, each cell line was treated with HB at its IC_50_ concentration and SD2 at its IC_50_ concentration. Additionally, selected combination treatments were applied: 0.2- and 0.8-fold of the HB IC_50_ concentration with 0.8- and 0.2-fold of the nanoceria IC_50_ concentration, respectively. Also, a combination of both compounds at their IC_50_ concentrations was tested. The untreated cells served as a control.

After 24 or 72 h of treatment, the medium was removed, and the cells were washed with 1× PBS. Following the manufacturer’s instructions, the cells were additionally washed, resuspended in Annexin Buffer and stained with Annexin V and PI at a 1:2 ratio, with the volume adjusted based on the cell count. The samples were then incubated for 15 min in the dark at room temperature, followed by the addition of another 100 µL of Annexin Buffer before measurement on a BD FACS Canto II instrument and analysis using FlowJo v10 software.

### 4.9. Spheroid Growth Analysis

In addition to the established standard 2D cultivation of the cell lines MG-63, Saos-2, and hFOB 1.19, 3D cultures of each cell line were established to conduct preliminary tests of treatment effects in three-dimensional growth models in the form of spheroids by the liquid layer or floating cells method [42]. Prior to the cell cultivation, a hot, sterile 1.5% agarose gel was poured in a 96-well plate, followed by cell seeding with a density of 5000 cells/well. Spheroids were usually formed within 24 h after the seeding when bright-field images of all spheroid replicas were acquired with a 10× objective of an EVOS M5000 imaging system. Afterwards, the same treatment regime was performed as previously performed in the 2D cell culture for flow cytometry analysis. Spheroids were imaged again at 24 h and 72 h after the treatment.

The spheroid area, as a morphological descriptor, was analyzed using ImageJ v0.5.7 image-processing software to determine the growth rate. The measurement of the spheroid area from transmitted-light images is described as a classical method to evaluate the activity of antiproliferative compounds in 3D models [43]. The growth rates at 24 h and 72 h were calculated for each spheroid relative to the spheroid area before the addition of treatments. The average growth rate at the HB and SD2 IC_50_ concentrations and combined treatments was normalized to the average growth rate of the untreated spheroids (controls) for each executed experiment.

### 4.10. Statistical Analysis

Statistical analyses were conducted using GraphPad Prism software (version 10.4.1). The differences between the treatment groups were estimated using one-way and two-way analyses of variance (ANOVA), followed by Tukey’s post hoc test. A *p*-value of less than 0.05 was considered statistically significant, with significance levels denoted as * *p*  <  0.05, ** *p*  <  0.001, *** *p*  <  0.001, and **** *p* < 0.0001.

## 5. Conclusions

In our work we aimed to investigate the single and combined treatment effects of HB and SD2 on selected osteosarcoma cell lines and corresponding healthy cell controls in 2D and 3D (spheroid) microenvironments. The presented results emphasize the importance of dose, exposure time, and cellular context in determining the effects of HB and SD2. While HB demonstrated clear antitumor activity through cytotoxicity and ROS induction, its combination with redox-active nanoparticles, such as SD2, could either enhance or attenuate these effects depending on the ratio and cell type. We found that the combined treatment of 20% HB + 80% SD2 produced replicable and reliable antitumor effects in both osteosarcoma cell lines. This can be explained by specific biochemical interactions and microenvironment generation of HB and SD2 that were previously described in independent studies. The tumor selectivity in this study was more evident in the 3D models, which better replicated the in vivo tissue structure and treatment diffusion limitations. However, one of the key limitations of this study was the exclusive use of established cell lines for both the 2D and 3D (spheroid) models. While these models provide valuable initial insights into the cytotoxic and redox effects of HB and SD2, they do not fully replicate the complexity of the tumor microenvironment or the heterogeneity observed in patient tumors. Cell lines often exhibit genetic and phenotypic drift from primary tumors and may not accurately represent inter-patient variability. Therefore, future studies should consider incorporating patient-derived organoids or spheroids, which more closely mimic the architecture, cellular diversity, and treatment responses of tumors in vivo. These advanced models would provide a more physiologically relevant platform for evaluating the therapeutic potential and selectivity of HB–SD2 combination treatments. The observed complexity of antitumor activities of HB and SD2 as single treatments and combined underscores the need for further mechanistic studies, particularly at the transcriptomic and proteomic levels, to delineate the exact pathways modulated by HB–SD2 co-treatments that drive the explicit and desired biological effect.

## Data Availability

The original contributions presented in this study are included in the article/Supplementary Material. Further inquiries can be directed to the corresponding authors.

## Supplementary Materials

The following supporting information can be downloaded from https://www.mdpi.com/article/10.3390/ijms26209837/s1.
