# Supplementary material for: Targeting Osteosarcoma: The Dual Action of Halogenated Boroxine and Cerium Oxide Nanoparticles
Source: Int J Mol Sci. 2025 Oct 10;26(20):9837. doi: 10.3390/ijms26209837 (PMC12564822; doi:10.3390/ijms26209837)
Supplement: Supplementary file 1 [file ijms-26-09837-s001.zip › ijms-3731140-supplementary.pdf]

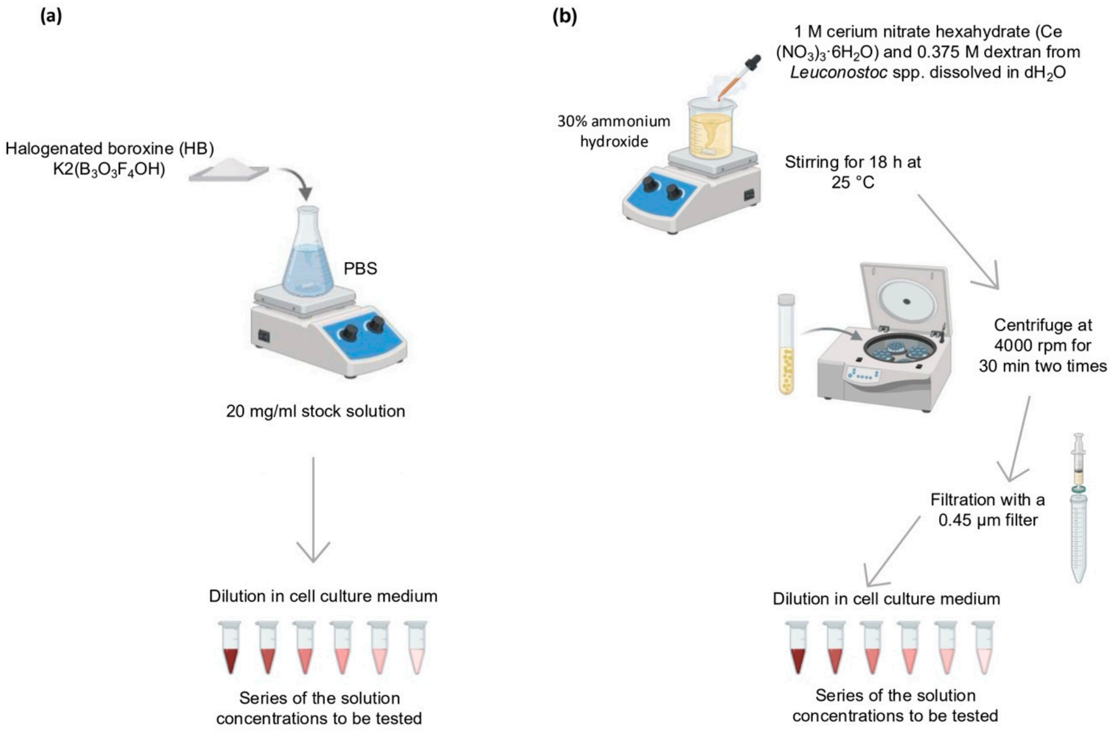

Supplementary Figure S1. Schematic overview of the preparation processes of (**a**) halogenated boroxine (HB) and (**b**) dextran-coated cerium oxide nanoparticles (SD2).

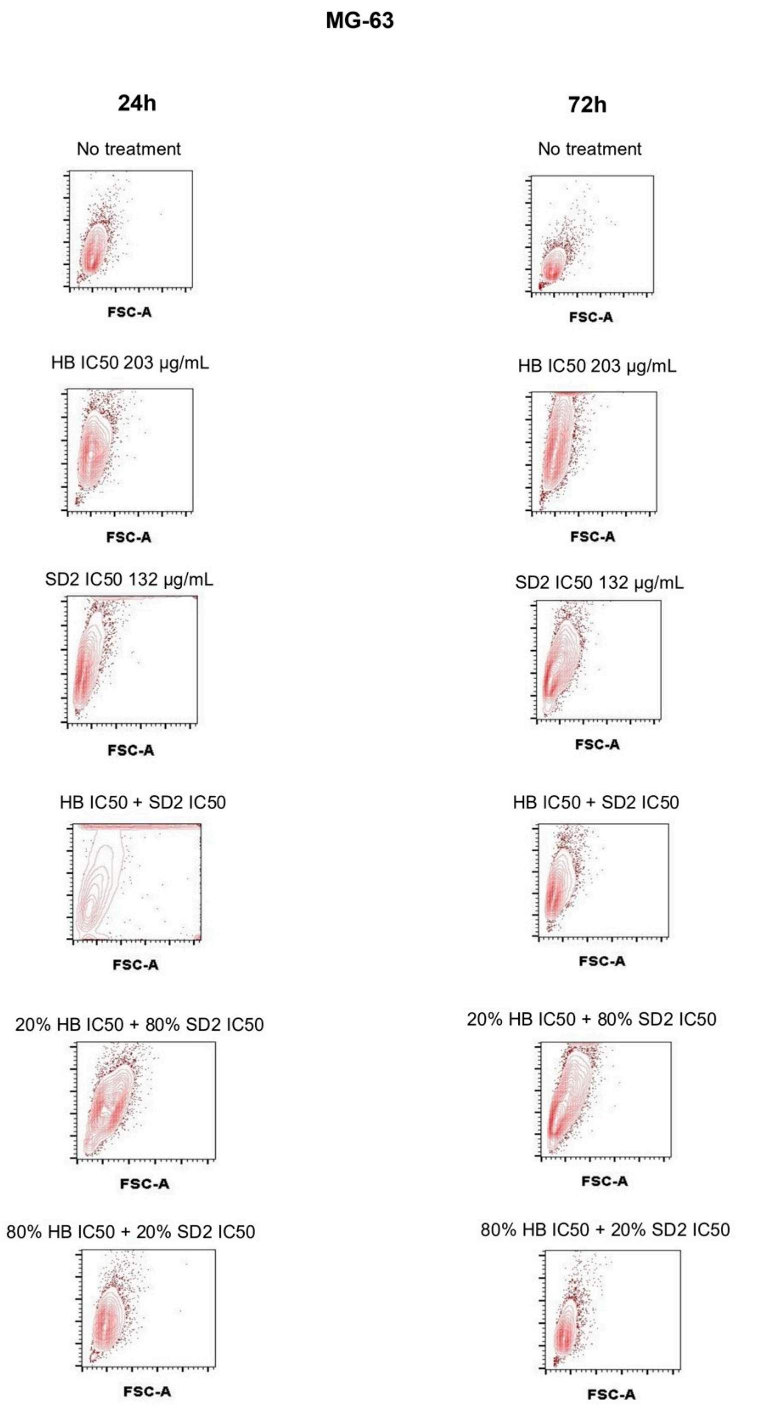

Supplementary Figure S2. FSC-A flow cytometry profiles of MG-63 cells under untreated, single, and combined treatment conditions at 24 h and 72 h.

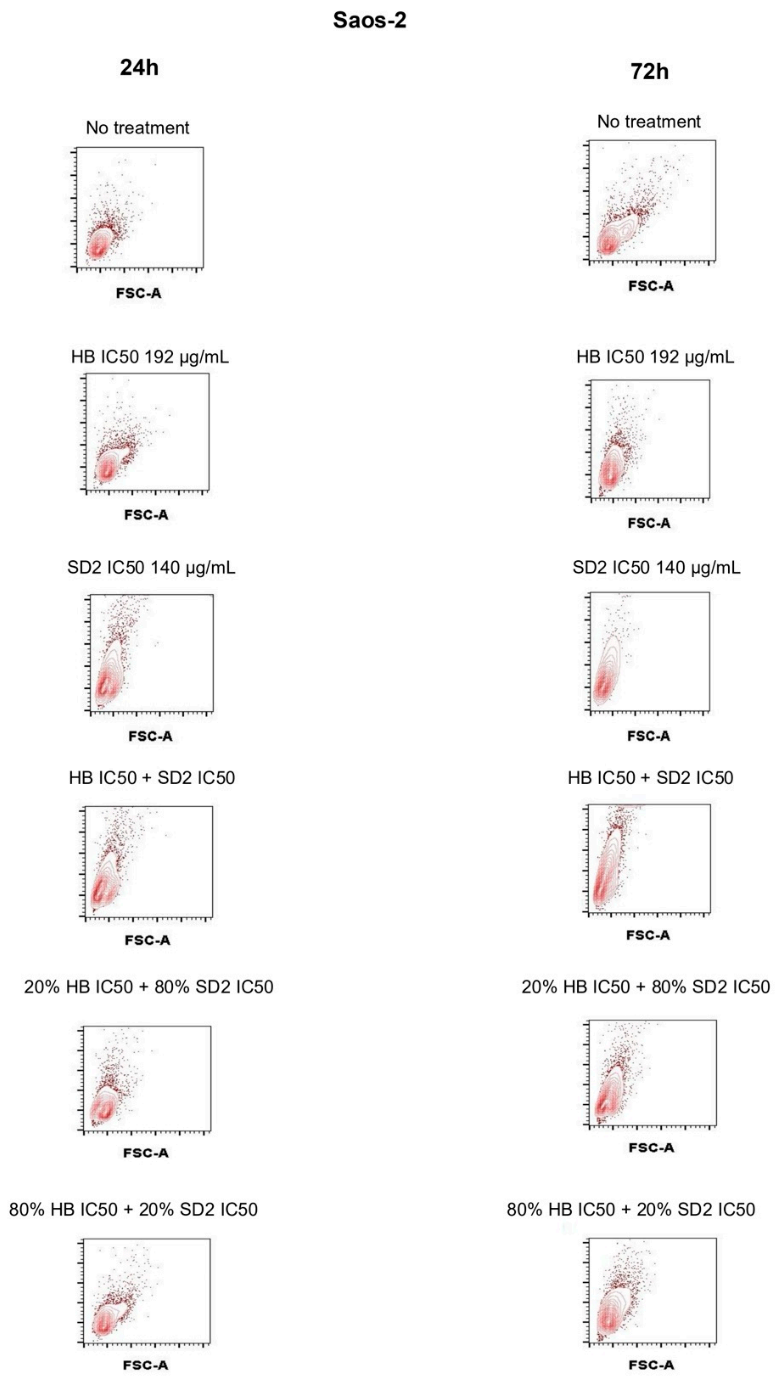

Supplementary Figure S3. FSC-A flow cytometry profiles of Saos-2 cells under untreated, single, and combined treatment conditions at 24 h and 72 h.

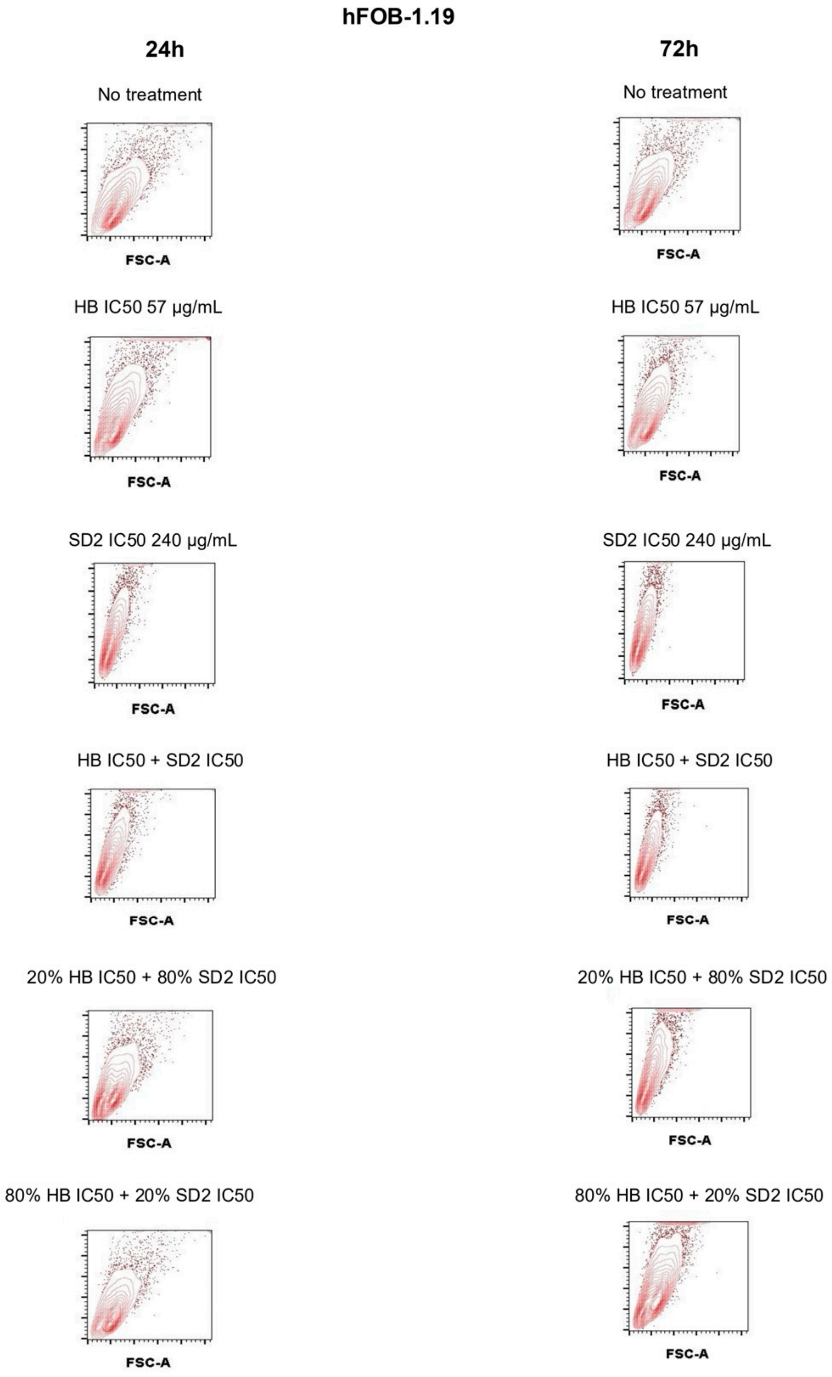

Supplementary Figure S4. FSC-A flow cytometry profiles of hFOB 1.19 cells under untreated, single, and combined treatment conditions at 24 h and 72 h.
